# Incidence and risk factors of congenital heart disease in Qingdao: a prospective cohort study

**DOI:** 10.1186/s12889-021-11034-x

**Published:** 2021-06-02

**Authors:** Xiao Jin, Wei Ni, Guolan Wang, Qin Wu, Jun Zhang, Guoju Li, Na Jiao, Wenjing Chen, Qing Liu, Li Gao, Quansheng Xing

**Affiliations:** 1 Qingdao University, Qingdao, Shandong Province China; 2 Qingdao Women and Children’s Hospital, Qingdao University, Qingdao, Shandong Province, No.6 Tongfu Road, Qingdao, 266000 China; 3 Qingdao Maternal and Child Health Care and Family Planning Service Center, Qingdao University, Qingdao, Shandong Province China

**Keywords:** Epidemiologic studies, Congenital heart defects, Incidence, Regression analysis, Risk factors

## Abstract

**Background:**

Many studies have been conducted to assess the incidence of congenital heart disease (CHD). However, results were greatly inconsistent among these studies with a broad range of findings.

**Methods:**

A prospective census-based cohort study was conducted in Qingdao, China, from August 1, 2018 to April 30, 2019. All of the local registered pregnant women were continuously investigated and followed from 15 to 20 weeks of gestation to delivery, tracking the CHD cases in both the fetal and neonatal stages. A logistic regression model was applied to assess the association between CHD and possible risk factors.

**Results:**

The positive rate of prenatal CHD screening was 14.36 per 1000 fetuses and the incidence of CHD was 9.38 per 1000 live births. Results from logistic regression indicated that, living in the countryside (odds ratio, (OR): 0.771; 95% confidence interval, (CI): 0.628–0.946) and having a childbearing history (OR: 0.802; 95%CI: 0.676–0.951) were negatively associated with CHD. However, twin pregnancy (OR: 1.957, 95% CI: 1.245–3.076), illness in the first trimester (OR: 1.306; 95% CI: 1.048–1.628), a family history of CHD (OR: 7.156; 95% CI: 3.293–15.552), and having a child with a birth defect (OR: 2.086; 95% CI: 1.167–3.731) were positively associated with CHD.

**Conclusion:**

CHD is a serious health problem in Qingdao. The CHD incidence found in this study was similar to existing research. The positive rate of prenatal CHD screening was higher than the incidence of neonatal CHD. Moreover, CHD risk factors were identified in our study, and our findings may have great implications for formation CHD intervention strategies.

**Supplementary Information:**

The online version contains supplementary material available at 10.1186/s12889-021-11034-x.

## Introduction

Congenital heart disease (CHD) is typically defined as a structural abnormality of the heart and/or great vessels during the embryonic period, also known as congenital heart malformation. During the embryonic development period (especially within the first 2–3 months of pregnancy), local anatomical structure abnormalities caused by the formation of the heart and large blood vessels occur, or there is a failure to close the channels that should be automatically closed after birth (normal in the fetus). This is referred to as CHD [1]. The categories of severity of CHD are as follows: severe CHD includes all those with cyanotic heart disease (d-transposition of the great arteries, tetralogy of fallot, including pulmonary atresia and absent pulmonary valve, hypoplastic right heart, hypoplastic left heart, single ventricle, double outlet right ventricle, truncus arteriosus, total anomalous pulmonary venous connection, and critical pulmonic stenosis) and acyanotic lesions (atrioventricular septal defect, large ventricular septal defect, large patent ductus arteriosus, critical or severe aortic stenosis, severe pulmonic stenosis, and critical coarc); moderate CHD includes mild or moderate aortic stenosis or aortic incompetence, moderate pulmonic stenosis or incompetence, noncritical coarc, large atrial septal defect, and complex forms of ventricular septal defects; and mild CHD includes small ventricular septal defect, small patent ductus arteriosus, mild pulmonic stenosis, bicuspid aortic valve without aortic stenosis or aortic incompetence, and small or spontaneously closed atrial septal defects [2]. Since the beginning of the twenty-first century, CHD has been the most frequent form of congenital anomaly found in newborn infants around the globe, accounting for one-third of all anomalies with high mortality [3]. An updated systematic review and meta-analysis of 260 studies revealed that between 1970 and 2017, the prevalence of CHD globally increased by 10% every 5 years [4].

To date, many studies have been conducted to assess the prevalence of CHD and its associated factors. However, results were greatly inconsistent among these studies with a range from 4/1000 to 50/1000 [2]. In North America, a prevalence of 6.9 per 1000 live births (95% CI: 6.7–7.1) was reported in CHD cases [3]. In China, CHD was identified in 1103 neonates, with an updated overall prevalence of 8.98 per 1000 in 18 hospitals, including the eastern and western regions [5]. However, recently researchers described the prevalence of circulatory system malformations as 3.6/1000 births, based on a prefecture-wide hospital-based birth cohort at the beginning of 12 weeks gestation in Japan [6]. Genetic and environmental factors are the two most important factors affecting the risk of CHD [7, 8]. It was considered that the difference of CHD incidence in different areas may be attributed to geographical, demographic, and socio-economic variations [9]. Strikingly, an increasing body of data has revealed the different incidences of CHD in different areas. We think that this phenomenon might be related to study methods in addition to genetic and environmental factors, because bias in the sampling monitor could result in inaccurate data. Qingdao is situated in eastern China, and has a mid-temperate continental monsoon climate with an annual average temperature of 12.7 °C. In our study, we aimed to identify the incidence and risk factors of CHD in Qingdao. We conducted a municipal census and prospective cohort study, involving all of the pregnant women in Qingdao.

## Methods

### Study design and data collection

A birth cohort was conducted enrolling 64,763 registered pregnant women at 15–20 weeks of gestation from August 1, 2018 to April 30, 2019, on the overall city-wide level. This study was carried out by the Qingdao Municipal Center for Birth Defect Control and Prevention (QMCBDCP) and Qingdao Women and Children’s Hospital (QWCH). All of the registered pregnant women were continuously followed from the fetal stage to the neonatal stage to track CHD cases. The data collection tool was developed according to the following steps. First, we designed a questionnaire by consulting the literature to investigate the characteristics of pregnant women and their partners, such as demographic information, pregnancy information, health status and other potential risk factors such as maternal dietary intake during pregnancy. Next, the questionnaires were sent to prenatal screening hospitals throughout the city and filled out by all of the pregnant women getting the Down’s screening at 15–20 weeks of gestation. In this study, we focused on CHD from the fetal stage to the neonatal stage. Two screenings for CHD were performed on enrolled pregnant women. One was performed on fetuses during 20–28 weeks of gestation, and another was performed on newborns at birth. If the fetus was prenatally screened with fetal CHD, the family would be informed about the condition and provided with medical counseling by a multidisciplinary team in QMCBDCP and QWCH. The prenatal cases of CHD reconfirmed the diagnosis again before discharge in the neonatal stage. For each pregnant woman, we continuously the followed results of these two screenings as well as the pregnancy outcomes, which were obtained by telephone follow-up to enrolled pregnant women and electronic medical records in medical institutions. In order to ensure that all targeted pregnant women were enrolled, our study included all relevant institutions such as prenatal screening and delivery hospitals in the maternal and child health management network. For the accuracy of information and in order to minimize bias among hospitals, investigators, and echocardiographers, we conducted systematic training for the staff committed to the survey as well as routine sampling checks. In addition, the identity card numbers of enrolled pregnant women were used as the unique identification code linking the information through continuous stages.

### Definitions

In our study, we identified all of the CHD cases, including fetuses and newborns based on whether there was a structural abnormality of the heart and/or great vessels. All types of CHD were coded according to ICD-11. All fetal CHD cases were screened by fetal ultrasound scan, and neonatal CHD cases were screened and diagnosed by pulse oximetry screening, clinical observation, physical examination, echocardiography, cardiac catheterization and magnetic resonance angiography (MRA) as needed [10–13]. A fractional (as opposed to functional) oxygen saturation of ≥94% was accepted as normal [14–16]. The positive rate of prenatal CHD screening was calculated as the total number of fetal heart structure abnormalities in the cohort divided by sum of fetuses screened in the same period. The incidence rate in newborns was calculated as the total number of neonatal CHD in the cohort divided by the sum of neonates in the same period. The false negative rate was equal to the number of false negatives divided by the sum of true positives and false negatives. In our study, pre-pregnancy body mass index (BMI) was categorized according to the World Health Organization adult criteria. BMI < 18.5 kg/m^2^ was considered lean, BMI between 18.5 and 24.9 kg/m^2^ was considered healthy, and BMI ≥ 25 kg/m^2^ was considered overweight or obese. Education levels were categorized as low (received no education, primary school, or secondary school), medium (high school or college/university), and high (postgraduate or above). We collected information on dietary patterns (a well-balanced diet (including both vegetables and meat, a diet with less vegetables, and a diet with less meat) of pregnant women before pregnancy. The history of illness in first trimester, which included hypertension, diabetes, anemia, cold, fever, thyroid disease, rubella virus infection, hepatitis B, genitourinary system diseases and immune system diseases was recorded and reflected the health status of the pregnant women. A history of having a child with birth defects meant that the pregnant woman gave birth to one or more children with birth defects before this pregnancy. A family history of birth defects referred to birth defects of immediate relatives of the couple. A family history of CHD referred to CHD of immediate relatives of the couple. Husband smoking before pregnancy was categorized at four levels: does not smoke, smokes less than 10 cigarettes a day, smokes between 10 and 20 cigarettes a day, and smokes more than 20 cigarettes a day. Atrial septal defects within oval fossa (sinus venosus defects, interatrial communication through the coronary sinus orifice and other specified congenital anomalies of atrial septum were not included), patent foramen ovale, and patent ductus arteriosus among fetuses and neonates < 28 days of life were excluded because they are normal fetal and neonatal findings.

This study was approved by the Ethics Commission of Qingdao Women and Children’s Hospital (QFFLL-KY-2020-11) and written informed consent was obtained from involved patients prior to enrollment. To minimize bias among hospitals, investigators and echocardiographers, we provided systematic training for the staff committed to the survey.

### Data statistical analysis

In order to detect the independent factors of CHD, univariate and multivariable analyses were performed. Chi-square test were first conducted with a total of 16 factors between the CHD and no CHD groups. All of the factors are listed in Table 1, revealing their distribution. Univariate logistic regression analysis also was performed. A multivariable regression model was then performed with significant factors selected by univariate analysis to tease out the independent factors for CHD. The association between independent factors and CHD was quantified by odds ratio (OR). Meanwhile, model selection was performed by excluding some uncertain data or changing the definition of a major variable of interest or outcome.

**Table 1:** Characteristics and distribution for risk factors between CHD and no CHD group^a^

Risk factors	Total	CHD n (%)	No CHD n (%)	χ2 value	*P* value
Twin pregnancy					
No	63,590	582 (0.92%)	63,008(99.08%)	7.801	**0.005**
Yes	1173	20(1.71%)	1153(98.29%)		
Maternal age (years)					
< 35	51,651	484(0.94%)	51,167(99.06%)	1.560	0.692
≥ 35	13,112	118(0.90%)	12,994(99.10%)		
BMI (kg/m^2^)					
< 18.5	5269	61(1.16%)	5208(98.84%)	−1.162	0.245
18.5–24.9	37,202	361(0.97%)	36,841(99.03%)		
≥ 25	11,603	108(0.93%)	11,495(99.07%)		
Living location					
City	40,274	424(1.05%)	39,850(98.95%)	8.334	**0.004**
Countryside	15,324	120(0.78%)	15,204(99.22%)		
Assisted reproduction					
No	54,827	531(0.97%)	54,296(99.03%)	3.704	0.054
Yes	789	13(1.65%)	776(97.25%)		
Maternal educational level					
Low	11,957	92(0.77%)	11,865(99.23%)	−3.274	**0.001**
Medium	40,605	409(1.01%)	40,196(98.99%)		
High	3043	43(1.41%)	3000(98.59%)		
Dietary patterns					
Well-balanced diet	47,409	465(0.98%)	46,944(99.02%)	−0.114	0.909
Eat less meat	6440	61(0.95%)	6379(99.05%)		
Eat less vegetables	1747	18(1.03%)	1729(98.07%)		
Husband age (years)					
< 35	38,489	395(1.03%)	38,095(98.97%)	2.892	0.089
≥ 35	17,081	149(0.87%)	16,932(99.13%)		
Number of previous pregnancies					
0	17,500	189(1.08%)	17,311(98.92%)	−2.215	**0.027**
1	20,106	201(1.00%)	19,905(99.00%)		
> 1	18,013	153(0.85%)	17,860(99.15%)		
Fertility history					
0	26,021	288(1.11%)	25,733(98.89%)	8.615	**0.003**
≥ 1	29,598	255(0.86%)	29,343(99.14%)		
History of having a child with birth defects					
No	55,015	531(0.97%)	54,484(99.03%)	6.449	**0.011**
Yes	604	12(1.99%)	592(98.01%)		
History of illness in the first trimester					
No	47,776	446(0.93%)	47,330(99.07%)	6.940	**0.008**
Yes	7844	98(1.25%)	7746(98.75%)		
Family history of birth defect					
No	55,222	534(0.97%)	54,688(99.03%)	9.746	**0.002**
Yes	398	10(2.51%)	388(97.49%)		
Family history of CHD					
No	55,521	537(0.97%)	54,984(99.03%)	38.012	**< 0.001**
Yes	99	7(7.07%)	92(92.93%)		
History of folic acid intake					
Does not eat folic acid	3423	21(0.61%)	3402(99.39%)	−2.279	**0.022**
Eating folic acid 3 months before after pregnancy	37,683	364(0.97%)	37,319(99.03%)		
Eating folic acid 6 months before and after Pregnancy	14,514	159(1.10%)	14,355(98.90%)		
History of husband smoking (cigarettes)					
0	34,101	355(1.04%)	33,746(98.96%)	−1.641	0.101
< 10	10,674	87(0.82%)	10,587(99.18%)		
10–19	7059	68(0.96%)	6991(99.04%)		
≥ 20	3786	34(0.90%)	3752(99.10%)		

^a^For each factor, except for counts of its levels listed in the table, the rest was the count of missing data

A *p* value less than 0.05 (two-tailed) was considered statistically significant. The missing data for each factor was removed for both univariate and multivariable analysis, and the rate of missing data ranged from 0 to 16.5%. Analyses were performed using SPSS software version 22.0.

## Results

### Data characteristics

A total of 64,763 pregnant women were enrolled in the cohort and followed up from 15 to 20 weeks of gestation to birth. The follow-up time was approximately 9–29 weeks.

**Fig. 1:**
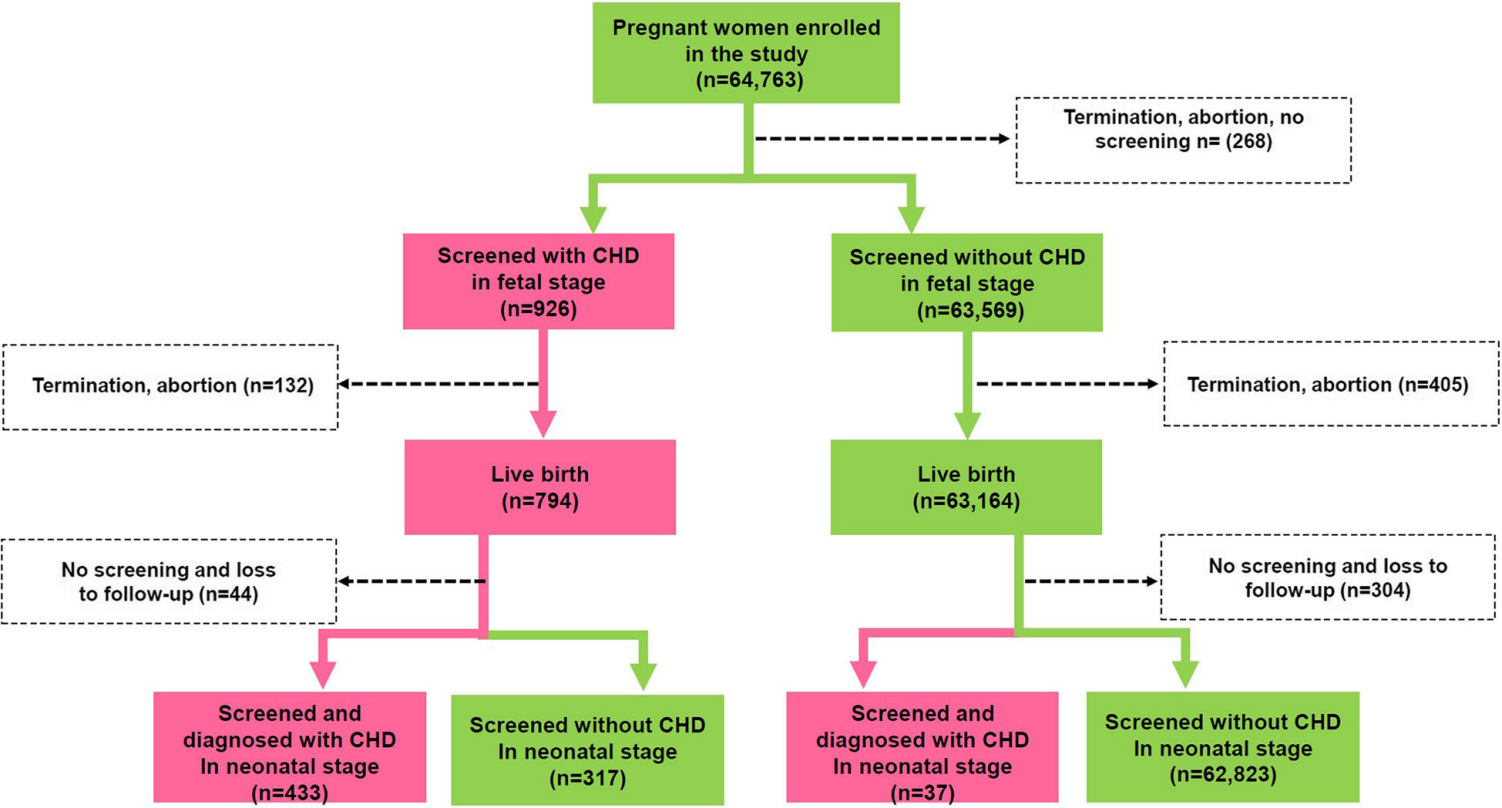
The distribution of screened CHD cases from the fetal to the neonatal stage

Among all the pregnant women, we were able to identify 805 (1.24%) pregnancies that ended with termination of pregnancy, abortion, or stillbirth, 63,958 (98.76%) pregnancies in which newborns were delivered, and 348 (0.54%) pregnancies in which the outcome could not be tracked due to failure to follow-up. As shown in Fig. 1, 268 pregnancies were terminated before the first stage of prenatal screening, and 926 CHD cases were screened in the fetal stage, with a positive rate of prenatal CHD screening of 14.36 per 1000 fetuses (926/64,495). Other than cases that ended in termination, abortion, 794 fetal cases of CHD were delivered. Among these births, 433 were screened and diagnosed with neonatal CHD in the neonatal stage, 317 were screened but not diagnosed with neonatal CHD, and 44 were not screened and failed to follow up. Among the 63,164 newborns that were screened but not diagnosed with CHD in the fetal stage, 37 of them were screened with neonatal CHD after delivery. Overall, CHD incidence was 9.38 per 1000 live births (602/64,147). The false negative rate was 6.15% (37/602).

**Fig. 2:**
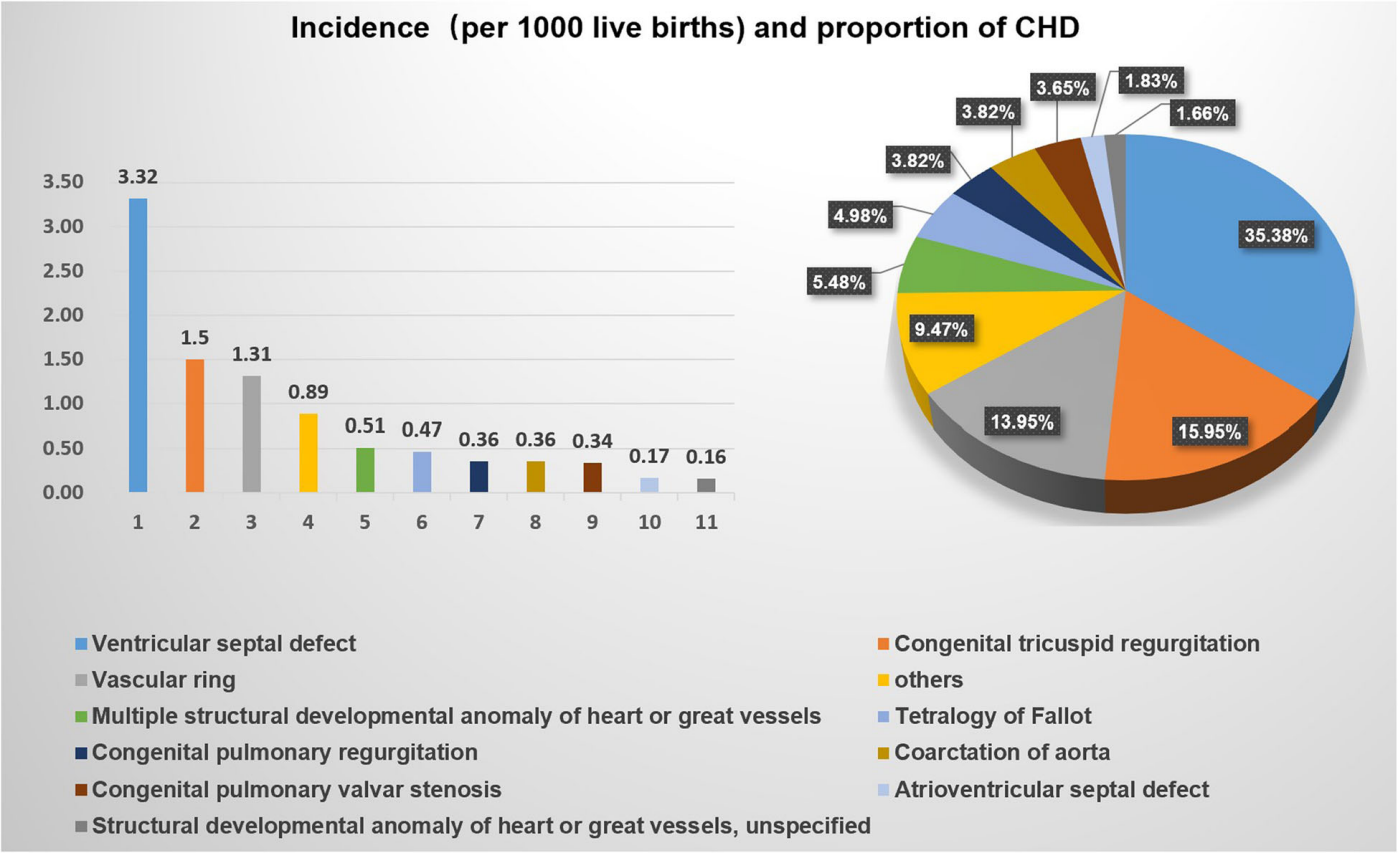
The incidence and proportion of CHD

In our study, all of the CHD cases, including fetuses and newborns, were classified into 30 categories according to ICD-11. As shown in Table 2, the predominant types of CHD in the fetal stage were congenital tricuspid regurgitation (31.64%), ventricular septal defect (31.10%), vascular ring (8.53%), and congenital pulmonary regurgitation (5.72%). In the neonatal stage, the predominant types of CHD were ventricular septal defect (42.13%), congenital tricuspid regurgitation (19.36%), vascular ring (16.81%), and congenital pulmonary regurgitation (4.89%). The main types and incidence of CHD screened and diagnosed are depicted in Fig. 2. As shown in Table 3, the types of CHD without abnormalities after delivery mainly included congenital tricuspid regurgitation (62.78%), ventricular septal defect (23.03%), and congenital pulmonary regurgitation (9.46%) in prenatal screening. In the newborn screening stage, the types of missed CHD mainly included ventricular septal defect (54.05%), vascular ring (13.51%), congenital tricuspid regurgitation (5.41%), and atrial septal defect (5.41%).

**Table 2:** Proportion of different types of CHD in both of the fetal and neonatal stage according to ICD-11

Congenital heart disease	ICD-11 code	Fetus n (%)	Termination n (%)	Newborns n (%)
Transposition of the great arteries	LA85.1	7(0.76%)	7(5.30%)	0(0.00%)
Double outlet right ventricle	LA85.2	4(0.43%)	3(2.27%)	1(0.21%)
Common arterial trunk	LA85.4	1(0.11%)	0(0.00%)	2(0.43%)
Partial anomalous pulmonary venous connection	LA86.21	1(0.11%)	2(1.52%)	1(0.21%)
Congenital tricuspid regurgitation	LA87.00	293(31.64%)	5(3.79%)	91(19.36%)
Ebstein malformation of tricuspid valve	LA87.0Y	1(0.11%)	1(0.76%)	0(0.00%)
Congenital mitral regurgitation	LA87.10	7(0.76%)	0(0.00%)	2(0.43%)
Atrioventricular septal defect	LA87.20	11(1.19%)	9(6.82%)	2(0.43%)
Tetralogy of Fallot	LA88.2	29(3.13%)	20(15.15%)	10(2.13%)
Ventricular septal defect	LA88.4	288(31.10%)	15(11.36%)	198(42.13%)
Hypoplastic right heart syndrome	LA88.Y	5(0.54%)	4(3.03%)	1(0.21%)
Hypoplastic left heart syndrome	LA89.3	3(0.32%)	3(2.27%)	1(0.21%)
Functionally univentricular heart	LA89	6(0.65%)	6(4.55%)	0(0.00%)
Congenital pulmonary valvar stenosis	LA8A.00	27(2.92%)	3(2.27%)	19(4.04%)
Congenital pulmonary regurgitation	LA8A.01	53(5.72%)	0(0.00%)	23(4.89%)
The dysplastic pulmonary valve	LA8A.0Y	2(0.22%)	1(0.76%)	1(0.21%)
Anomalous origin of the pulmonary artery from aortic artery	LA8A.Y	1(0.11%)	1(0.76%)	0(0.00%)
Congenital pulmonary atresia	LA8A.1	5(0.54%)	4(3.03%)	1(0.21%)
Congenital aortic regurgitation	LA8A.21	2(0.22%)	0(0.00%)	1(0.21%)
Bicuspid aortic valve	LA8A.22	1(0.11%)	0(0.00%)	1(0.21%)
Coarctation of aorta	LA8B.21	23(2.48%)	1(0.76%)	22(4.68%)
Interrupted aortic arch	LA8B.22	2(0.22%)	2(1.52%)	0(0.00%)
Vascular ring	LA8B.2Y	79(8.53%)	5(3.79%)	79(16.81%)
Congenital anomaly of great arteries including arterial duct, unspecified	LA8B.Z	6(0.65%)	0(0.00%)	0(0.00%)
Congenital coronary arterial fistula	LA8C.2	3(0.32%)	0(0.00%)	3(0.64%)
Divided left atrium	LA8G.0	1(0.11%)	1(0.76%)	0(0.00%)
Atrial septal defect	LA8E.1	4(0.43%)	2(1.52%)	4(0.85%)
Multiple structural developmental anomaly of heart or great vessels	LA8Y	32(3.46%)	27(20.45%)	6(1.28%)
Left ventricular cyst	LA8Y	0(0.00%)	0(0.00%)	1(0.21%)
Structural developmental anomaly of heart or great vessels, unspecified	LA8Z	29(3.13%)	10(7.58%)	0(0.00%)
Total	LA80-LA8Z	926(100.00%)	132(100.00%)	470(100.00%)

**Table 3:** Proportion of misdiagnosed and missed cases of CHD

Congenital heart disease	ICD-11 code	no CHD after delivery (Prenatal screening with heart abnormalities) n (%)	CHD after delivery (prenatal screening without heart abnormalities) n (%)
Common arterial trunk	LA85.4	0(0.00%)	1(2.70%)
Partial anomalous pulmonary venous connection	LA86.21	0(0.00%)	1(2.70%)
Congenital tricuspid regurgitation	LA87.00	199(62.78%)	2(5.41%)
Congenital mitral regurgitation	LA87.10	5(1.58%)	0(0.00%)
Atrioventricular septal defect	LA87.20	1(0.32%)	1(2.70%)
Tetralogy of Fallot	LA88.2	0(0.00%)	1(2.70%)
Ventricular septal defect	LA88.4	73(23.03%)	20(54.05%)
Hypoplastic left heart syndrome	LA89.3	0(0.00%)	1(2.70%)
Congenital pulmonary valvar stenosis	LA8A.00	3(0.95%)	1(2.70%)
Congenital pulmonary regurgitation	LA8A.01	30(9.46%)	0(0.00%)
Congenital aortic regurgitation	LA8A.21	1(0.32%)	0(0.00%)
Vascular ring	LA8B.2Y	0(0.00%)	5(13.51%)
Congenital anomaly of great arteries including arterial duct, unspecified	LA8B.Z	5(1.58%)	0(0.00%)
Atrial septal defect	LA8E	0(0.00%)	2(5.41%)
Multiple structural developmental anomaly of heart or great vessels	LA8Y	0(0.00%)	1(2.70%)
Left ventricular cyst	LA8Y	0(0.00%)	1(2.70%)
Total		317(100.00%)	37(100.00%)

### Factors associated with CHD

**Fig. 3:**
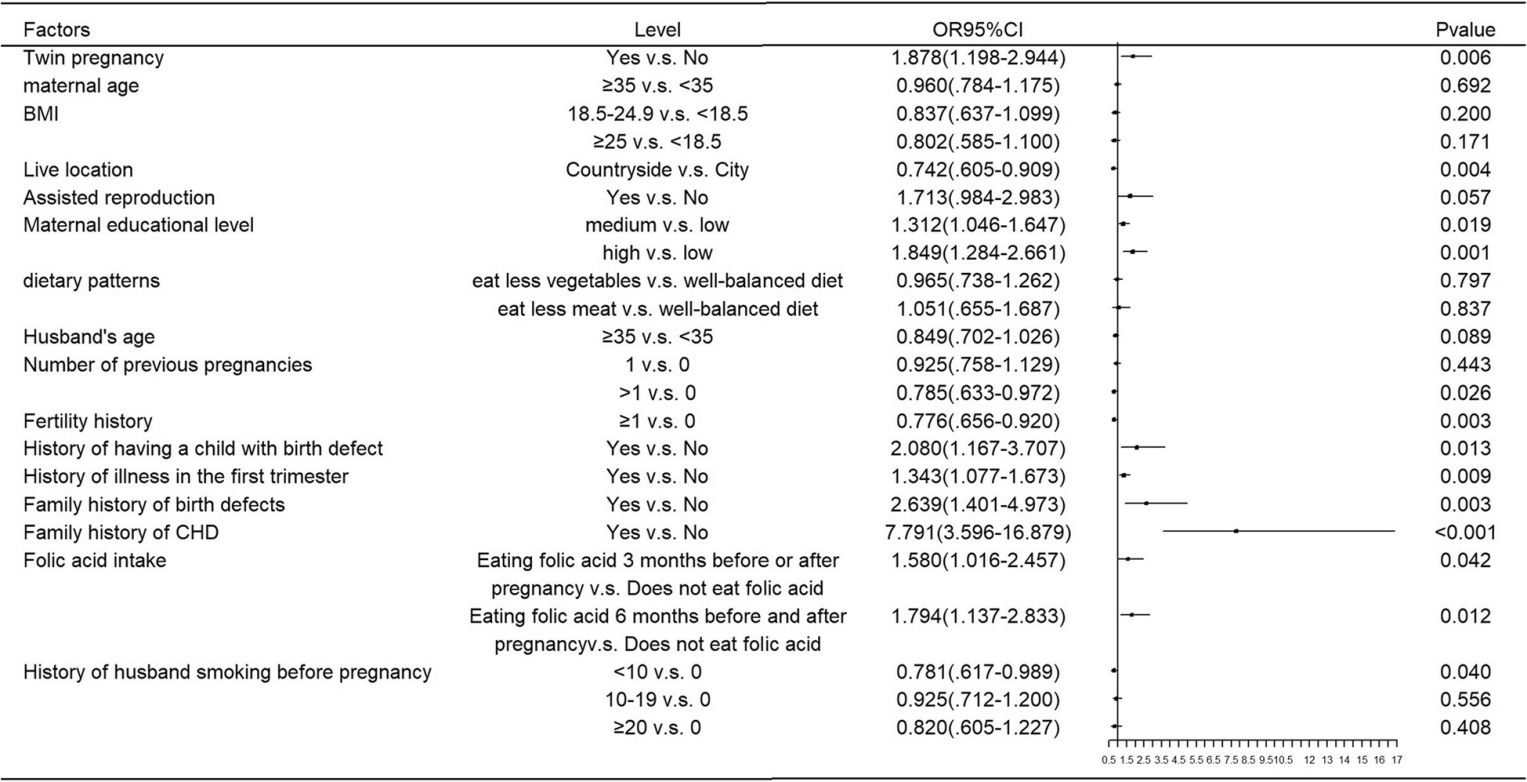
Results of univariate logistic regression analysis

**Fig. 4:**
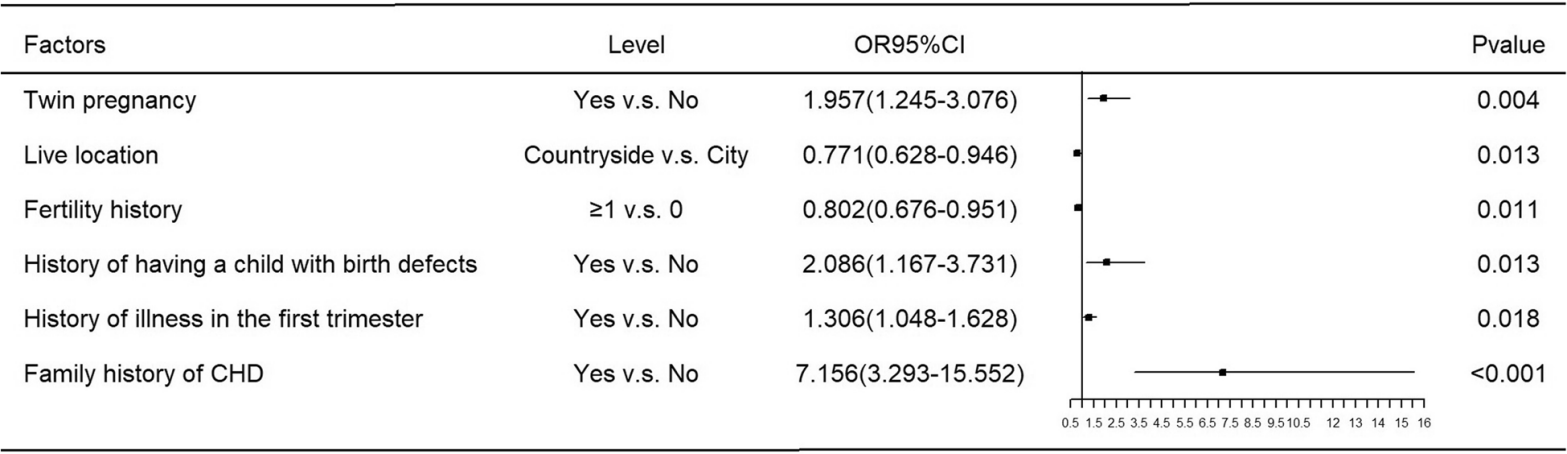
Results of multivariable logistic regression model

The results of univariate logistic regression analysis are show in Fig. 3. Twin pregnancy, educational level of pregnant women, living location, fertility history, the number of previous pregnancies, history of illness in the first trimester of pregnancy, history of having a child with birth defects, family history of birth defects and CHD, and history of folic acid intake were significantly related to CHD. Figure 4 summarizes the results of multivariable analysis, which showed that living in countryside (OR: 0.771; 95%CI: 0.628–0.946), having a childbearing history (OR: 0.802; 95%CI: 0.676–0.951), twin pregnancy (OR: 1.957, 95% CI: 1.245–3.076), illness in the first trimester (OR: 1.306; 95% CI: 1.048–1.628), family history of CHD (OR: 7.156; 95% CI: 3.293–15.552), and having a child with birth defects (OR: 2.086; 95% CI: 1.167–3.731) were independently associated with CHD.

Model selection was conducted to guarantee robust results. As shown in Additional files 1 and 2, the logistic regression analysis was performed by excluding some uncertain data to quantify the association between these significant factors detected by univariate analysis and CHD.

## Discussion

Previous studies from different areas in China have reported various incidences of CHD, which ranged from 6.87 to 76.00 per 1000 children [6, 17–22]. Compared with previous studies, our study could evaluate the incidence of CHD more accurately. It revealed that the incidence of CHD in the neonatal stage is close to existing research results, and that the positive rate of prenatal CHD screening is higher than that of the incidence of CHD in newborns. The main reasons are as follows: a high incidence of tricuspid regurgitation identified prenatally was likely to be a significant overestimate of true cardiac pathology, because at lesser levels this often reflects obstetric/placental /extra-cardiac fetal abnormality; pregnancies with complex CHD and some with mild CHD select termination; CHD is frequently misdiagnosed in the prenatal screening stage; and there were the cases we missed for the early clinical screening of neonates for CHD.

Our findings provided some important implications for CHD control and prevention. CHD is classified as simple, moderate and complex. As previous studies suggested, the variation in CHD incidence was attributed to minor CHD, but severe CHD remained stable [2]. Termination is not recommended for pregnant women with minor or mild fetal cardiac abnormality. Fetuses who were identified as CHD cases in the fetal stage can have a chance to improve their health with the development of tissues and organs to become a healthy newborn. A majority of the positive cases screened in the fetal stage, such as congenital tricuspid regurgitation and ventricular septal defects, are likely to be repaired by the time the baby is born. In prenatal screening, the false negative rate of prenatal screening was 6.15%. Ventricular septal defects were likely to be missed in the screening. However, this value might be overestimated, because these cases of ventricular septal defect were in the range of 3 mm, and they might be repaired in utero. Abortion and stillbirth should be of extreme concern for critical CHD fetuses. Most pregnant women and families in China lack knowledge of CHD and eventually choose to terminate with a resulting severe psychological burden. Several induction cases such as small ventricular septal defects could be effectively cured by surgery after birth, and they do not affect quality of life.

The risk factors associated with CHD were identified by the univariate, multivariable analyses. Results from the logistic regression model revealed that having a childbearing history and living in the countryside were negatively associated with CHD incidence, which may decrease the risk of CHD. In addition, a family history of CHD, history of illness in the first trimester, twin pregnancy, and history of having a child with birth defects before the current pregnancy were positively associated with CHD incidence, which may increase the risk of CHD. These results were mostly similar to those in previous studies [23–26]. Previous studies have pointed out that maternal education level is an independent factor affecting congenital heart disease [27, 28], but our study found that maternal education level and congenital heart disease have no significant correlation. Previous studies had shown that maternal folic acid supplementation is associated with a lower risk of congenital heart defects [29, 30]. In contrast, the results of our study showed no significant association between folic acid supplementation and CHD risks. This difference might be limited by the roughness of big data. We are collecting more comprehensive information about folic acid and analyzing the results.

There are several limitations in this study. First, several factors such as history of illness in the first trimester and dietary patterns were only summarized or subjective variables. In our study, all of these diseases were summarized as one variable aiming to assess the association between the health status of pregnant women and CHD, rather than between specific diseases and CHD. Moreover, according to this goal and design, there was no more information about the diseases obtained from enrolled pregnant women. In future research, we plan to obtain more detailed information on diseases to detect their association with CHD. The variable of dietary patterns was subjectively described by investigated pregnant women with no quantitative standard. As for the specific association between CHD and eating meat or vegetables and diseases, more information should be obtained in the future to evaluate their effect. Household income was an important variable to evaluate the association between socioeconomic level of the study population and CHD. We should increase the collection of household income information in the dataset. Through prenatal echocardiography and other examinations, we cannot determine whether fetal tricuspid regurgitation was caused by obstetric/ placental/ extra-cardiac fetal abnormality, which might overestimate the true cardiac pathology. CHD is possibly missed in the neonatal screening stage. In addition, missing data were inevitable in a large prospective census-based study, which may influence the accuracy of results. The missing data of each variable were removed for both univariate and multivariable analysis. Considering the large size of the enrolled the pregnant cohort (a total of 64,763women), there was still enough data to ensure that we obtained relatively precise results.

## Conclusions

CHD is a serious health problem in Qingdao. The CHD incidence found in this study was similar to existing research. The positive rate of prenatal CHD screening was higher than the incidence of neonatal CHD. Moreover, CHD risk factors were identified in our study, and our findings may have great implications for formation CHD intervention strategies.

## Supplementary Information

**Additional file 1.** Questionnaire.

**Additional file 2: Table 1 S1.** The results of multivariable regression analysis with the significant risk factors selected by univariate analysis. **Table 2 S2**. The results of multivariable regression analysis excluding the variable of number of previous pregnancies. **Table 3 S3.** The results of multivariable regression analysis excluding the variable of family history of birth defects.

## Data Availability

The datasets generated and/or analyzed during the current study are not publicly available due to the restrictions of the local ethics committee and institutional data security and privacy policies. The data access request needs institutional and ethics committee’s approval.

## Abbreviations

CHD: Congenital heart disease
OR: Odds ratio
MRA: Magnetic resonance angiography
VSD: Ventricular septal defect
CI: Confidence interval
QMCBDCP: Qingdao Municipal Center for Birth Defect Control and Prevention
QWCH: Qingdao Women and Children’s Hospital
BMI: Body mass index
SD: Standard deviation
IQR: Interquartile range
NSFC: The National Natural Science Foundation of China
