# Supplementary material for: Incidence and risk factors of congenital heart disease in Qingdao: a prospective cohort study
Source: BMC Public Health. 2021 Jun 2;21:1044. doi: 10.1186/s12889-021-11034-x (PMC8173734; doi:10.1186/s12889-021-11034-x)
Supplement: Supplementary file 1 — Additional file 1. Questionnaire. [file 12889_2021_11034_MOESM1_ESM.docx]

**Questionnaire**

1. Have you made an appointment for 4D color Doppler ultrasound? No, (appointment institution: date of appointment)

2. Have you had a pre pregnancy examination: No, Yes (examination institution: result: normal and abnormal)

3. Living location: city, countryside

4. Net height (barefoot): cm

5. Weight before pregnancy: kg

6. Maternal educational level: 1 illiterate, 2 primary school, 3 junior high school / technical school, 4 senior high school / technical secondary school, 5 junior college and Bachelor degree, 6 research and above

7. Pre pregnancy occupation:

8. In addition to this pregnancy, pregnancy times: abortion (spontaneous abortion times artificial abortion times), live birth (full-term live birth, premature birth (less than 37 weeks), stillbirth (stillbirth times neonatal death times within 7 days)

9. History of having a child with birth defects (such as congenital heart disease, syndactyly, polydactyly, cleft lip and palate, malformation of external ear, Down's syndrome, etc.): No, Yes: disease

10. History of illness in the first trimester: No, Yes: hypertension, diabetes, coronary heart disease, anemia, fever (>38℃) cold, thyroid disease, rubella virus infection, hepatitis B, urogenital system diseases, etc

11. History of drug in the first trimester:No, Yes: drug name, drug taking week, drug taking days

12. Family history of birth defect (such as congenital heart disease, syndactyly, polydactyly, cleft lip and palate, malformation of external ear, Down's syndrome, etc.): No, Yes: disease

13. History of folic acid intake: No, Yes(eating folic acid 3 months before pregnancy, eating folic acid 3 months after pregnancy)

14. Dietary patterns before pregnancy: Well-balanced diet, Eat less meat, Eat less vegetables

15. From the first half year of pregnancy to the first three months of pregnancy, are you exposed to the following factors in your life or work environment (multiple choices are allowed):

No, Yes (organic solvent (e.g. new decoration / paint), radiation (chest X-ray / CT, etc.), high temperature, noise and vibration, close contact with heavy metal (lead, mercury, etc.) pesticides such as cats and dogs, others)

16. Date of birth of husband:

17. History of husband smoking before pregnancy: No, Yes (cigarettes per day)
