# Supplementary material for: Incidence and risk factors of congenital heart disease in Qingdao: a prospective cohort study
Source: BMC Public Health. 2021 Jun 2;21:1044. doi: 10.1186/s12889-021-11034-x (PMC8173734; doi:10.1186/s12889-021-11034-x)
Supplement: Supplementary file 2 — Additional file 2: Table 1 S1. The results of multivariable regression analysis with the significant risk factors selected by univariate analysis. Table 2 S2. The results of multivariable regression analysis excluding the variable of number of previous pregnancies. Table 3 S3. The results of multivariable regression analysis excluding the variable of family history of birth defects. [file 12889_2021_11034_MOESM2_ESM.docx]

Table 1 S1 The results of multivariable regression analysis with the significant risk factors selected by univariate analysis

| Factors | Level | OR | 95%CI | *P* value |
| --- | --- | --- | --- | --- |
| Twin pregnancy | Yes v.s. No | 1.943 | 1.235-3.055 | 0.004 |
| Live location | Countryside v.s. City | 0.772 | 0.629-0.947 | 0.013 |
| Maternal educational level | low v.s. medium | 1.142 | 0.897-1.454 | 0.281 |
|  | low v.s. high | 1.472 | 0.999-2.165 | 0.051 |
| Number of previous pregnancies^[[1]](#footnote-1)^ | 1 v.s. 0 | 1.101 | 0.856-1.417 | 0.453 |
|  | >1 v.s. 0 | 1.031 | 0.736-1.338 | 0.958 |
| Fertility history^*^ | ≥1 v.s. 0 | 0.822 | 0.645-1.048 | 0.113 |
| History of having a child with birth defect | Yes v.s. No | 2.081 | 1.158-3.739 | 0.014 |
| History of illness in 1st trimester | Yes v.s. No | 1.297 | 1.040-1.618 | 0.021 |
| Family history of birth defects^[[2]](#footnote-2)^ | Yes v.s. No | 0.943 | 0.301-2.954 | 0.919 |
| Family history of CHD^†^ | Yes v.s. No | 7.394 | 1.868-29.262 | 0.004 |
| Folic acid intake | Eating folic acid 3 months before or after pregnancy v.s. Eating not folic acid | 1.466 | 0.942-2.283 | 0.090 |
|  | Eating folic acid 6 months before and after pregnancy v.s. Eating not folic acid | 1.583 | 0.999-2.508 | 0.050 |

Table 2 S2 The results of multivariable regression analysis excluding the variable of number of previous pregnancies

| Factors | Level | OR | 95%CI | P value |
| --- | --- | --- | --- | --- |
| Twin pregnancy | Yes v.s. No | 1.941 | 1.234-3.052 | 0.004 |
| Live location | Countryside v.s. City | 0.783 | 0.637-0.961 | 0.019 |
| Maternal educational level | medium v.s. low | 1.149 | 0.904-1.461 | 0.257 |
|  | high v.s. low | 1.477 | 0.999-2.168 | 0.051 |
| Fertility history^[[3]](#footnote-3)^ | ≥1 v.s. 0 | 0.839 | 0.704-0.999 | 0.048 |
| History of having a child with birth defect | Yes v.s. No | 2.072 | 1.158-3.708 | 0.014 |
| History of illness in 1st trimester | Yes v.s. No | 1.297 | 1.040-1.617 | 0.021 |
| Family history of birth defects | Yes v.s. No | 0.939 | 0.299-2.943 | 0.914 |
| Family history of CHD | Yes v.s. No | 7.473 | 1.889-29.565 | 0.004 |
| Folic acid intake | Eating folic acid 3 months before or after pregnancy v.s. Eating not folic acid | 1.469 | 0.944-2.288 | 0.088 |
|  | Eating folic acid 6 months before and after pregnancy v.s. Eating not folic acid | 1.587 | 0.998-2.514 | 0.052 |

Table 3 S3 The results of multivariable regression analysis excluding the variable of family history of birth defects

| Factors | Level | OR | 95%CI | *P* value |
| --- | --- | --- | --- | --- |
| Twin pregnancy | Yes v.s. No | 1.942 | 1.235-3.053 | 0.004 |
| Live location | Countryside v.s. City | 0.772 | 0.629-0.947 | 0.013 |
| Maternal educational level | low v.s. medium | 1.142 | 0.897-1.454 | 0.281 |
|  | low v.s. high | 1.471 | 0.999-2.165 | 0.050 |
| Number of previous pregnancies | 1 v.s. 0 | 1.101 | 0.856-1.417 | 0.453 |
|  | >1 v.s. 0 | 0.992 | 0.736-1.338 | 0.958 |
| Fertility history | ≥1 v.s. 0 | 0.822 | 0.645-1.048 | 0.114 |
| History of having a child with birth defect | Yes v.s. No | 2.079 | 1.158-3.734 | 0.014 |
| History of illness in 1st trimester | Yes v.s. No | 1.297 | 1.040-1.618 | 0.021 |
| Family history of CHD^[[4]](#footnote-4)^ | Yes v.s. No | 6.972 | 3.202-15.176 | <0.001 |
| Folic acid intake | Eating folic acid 3 months before or after pregnancy v.s. Eating not folic acid | 1.466 | 0.942-2.283 | 0.090 |
|  | Eating folic acid 6 months before and after pregnancy v.s. Eating not folic acid | 1.583 | 0.999-2.508 | 0.050 |

1. These two variables are statistically significant in Mann-Whitney U test, Chi-square test, and univariate analysis. We suspect that there is a correlation between number of previous pregnancies and fertility history. We performed Bivariate Correlations analysis, and found that These two variables are strongly correlated(Correlation coefficient is 0.656), and the result is significant(*P*<0.001). [↑](#footnote-ref-1)
2. These two variables are statistically significant in Mann-Whitney U test, Chi-square test, and univariate analysis, but the variable of family history of birth defects is not significant and the 95%CI of family history of CHD has a wide range of values. We suspect that there is a correlation between these two variables. We performed Bivariate Correlations analysis, and found that These two variables are strongly correlated(Correlation coefficient is 0.497), and the result is significant(P<0.001). [↑](#footnote-ref-2)
3. These two variables of number of previous pregnancies and fertility history are strongly correlated. We performed multivariable regression analysis excluding the variable of number of previous pregnancies, and found that the variable of fertility history becomes statistically significant. the variable of number of previous pregnancies is the uncertain data. [↑](#footnote-ref-3)
4. These two variables of family history of birth defects and family history of CHD are strongly correlated. We performed multivariable regression analysis excluding the variable of family history of birth defects, and found that the range of 95%CI of family history of CHD is narrower than before. The variable of family history of birth defects is the uncertain data. [↑](#footnote-ref-4)
